# A Case of Intraoperative Reproducible Transient Bradycardia During Total Hip Arthroplasty: Direct Activation of Parasympathetic Vagal Afferents From Bone

**DOI:** 10.1155/cria/5583839

**Published:** 2025-06-23

**Authors:** Yifan Li, Joseph Moskal, Maxine Lee

**Affiliations:** ^1^Carilion Clinic, 1906 Belleview Ave, Roanoke, Virginia 24014, USA; ^2^Virginia Tech Carilion School of Medicine, 2 Riverside Circle, Roanoke, Virginia 24016, USA; ^3^Department of Orthopaedics Surgery, Carilion Clinic, 1906 Belleview Ave, Roanoke, Virginia 24014, USA; ^4^Department of Anesthesiology, Carilion Clinic, 1906 Belleview Ave, Roanoke, Virginia 24014, USA

## Abstract

The Gallagher–Wilkinson reflex was first reported by Gallagher and Wilkinson in 2001, describing intraoperative transient and reproducible bradycardia and/or asystole during intramedullary reaming in patients undergoing total knee replacement. The reflex has been originally thought to result from an indirect mechanism: the elevated intramedullary pressure transmits to intrapelvic and intra-abdominal viscera, indirectly causing a vagally mediated reflex bradycardia. Other case reports have documented this reflex and discounted potential physiologic causes such as microemboli or primary cardiac arrhythmia. Several preclinical and clinical studies have demonstrated how the parasympathetic innervation of bone modulates bone remodeling. This paper presents a case of a patient who underwent a total hip replacement, experiencing intraoperative transient and reproducible bradycardia. We postulate a direct mechanism for the Gallagher–Wilkinson reflex: increased pressure from intramedullary reaming directly activates the parasympathetic nervous system in the bone axis leading to vagally-mediated bradycardia. This case offers an alternative explanation for the Gallagher–Wilkinson reflex and emphasizes the important need for additional research to better understand parasympathetic innervation in the bone axis.

## 1. Introduction

Degenerative hip arthritis is the leading cause of total hip arthroplasty (THA). In 2019, the annual volume of primary THA was over 250,000 cases and the volume is expected to increase by 24% every 5 years after 2020 [1]. Although THA offers the most definitive treatment for severe hip osteoarthritis refractory to medication and physical therapy, serious perioperative complications such as respiratory distress, fat embolism, and cardiac dysrhythmias have been reported [2–4]. We present the case of an 83-year-old female undergoing a THA who experienced transient and reproducible bradycardia/asystole during sequential reaming of the femur. This phenomenon has been previously reported as the Gallagher–Wilkinson reflex.

The Gallagher–Wilkinson reflex was first reported by Gallagher and Wilkinson after six patients undergoing total knee arthroplasty (TKA) experienced intraoperative transient and reproducible bradycardia/asystole which occurred when the surgeon applied high pressures to the distal femoral shaft while hammering [3]. Subsequent case reports by Wooster, et al. and Martinez, et al. described the Gallagher–Wilkinson reflex occurring during reaming of the femoral canal in surgical fixation of hip fractures [4, 5]. Gallagher and Wilkinson postulated an indirect mechanism for this reflex, that is, transient, and reproducible severe bradycardia/asystole resulted when high pressures applied to the femur were transmitted to the pelvis and intra-abdominal organs indirectly triggering this vagally-mediated bradycardia reflex [3]. In light of more recent research, we offer an alternate direct mechanism for the Gallagher–Wilkinson reflex as occurring when high mechanical pressures applied to the femur directly trigger the vagally-mediated reflexive bradycardia.

## 2. Case Presentation

An 83-year-old female with a medical history significant for hypertension, chronic atrial fibrillation (AF), hypothyroidism, and chronic lower back pain presented to the orthopedic clinic for evaluation of her severe left hip pain which was refractory to conservative measures. Medications included apixaban, diltiazem, levothyroxine, and acetaminophen. She ambulated with a walker and was physically deconditioned. The patient's history, physical exam, and left hip radiographs were consistent with severe, progressive arthritic changes. These findings were discussed with the patient, who elected to proceed with a left anterior THA.

Preoperative electrocardiogram demonstrated AF, rate 87 beats/min. Trans-thoracic echocardiography reported a normal left ventricular (LV) ejection fraction, normal global LV systolic function, mild concentric hypertrophy, a mildly dilated left atrium, and mild mitral regurgitation. She was mildly hyponatremic at 133 mmol/L, glucose was 102 mg/dL, and HbA1C was 5.8%. A physical exam on the morning of surgery revealed an elderly female, 162 cm, 64 kg, with stable vital signs, irregular heart rhythm consistent with AF, rate 99 beats/min, and clear lungs. Her American Society of Anesthesiologists physical status classification score was 3 given her cardiac dysrhythmia. Informed consent for a general anesthetic was obtained because inadequate time off apixaban did not ensure the safety of neuraxial anesthesia.

**Figure 1:**
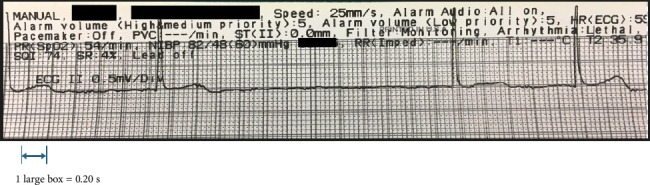
Example of intraoperative electrocardiogram tracing demonstrating the transition from atrial fibrillation to transient asystole when femoral reaming occurred and return to baseline after reaming stopped.

Induction of general anesthesia and intubation were uneventful. Medications administered at induction included propofol, lidocaine, rocuronium and fentanyl. Gentamicin and cefazolin were administered prior to skin incision. Approximately 1 hour after incision, the patient was noted to develop transient, recurrent episodes of bradycardia and asystole (Figure 1). Brief intermittent asystole was initially treated with boluses of dilute epinephrine (5 mcg/mL) until the anesthesiologist noted the association between the recurrent episodes of severe bradycardia/asystole with hammering of the femur. The surgeon was immediately alerted, hammering was paused, and the patient's baseline rhythm was restored. This was recognized as a vagally-mediated reflex and glycopyrrolate 0.2 mg was administered as prophylaxis against future similar events and the surgery proceeded to completion. The patient remained hemodynamically stable in the post-anesthesia care unit and was subsequently discharged to a skilled nursing facility for continued rehabilitation.

While in the skilled rehabilitation center, the patient received several visits from her medical team. She remained in rate-controlled, chronic AF with stable vital signs. She did not have cardiac-related complaints, nor did she exhibit any evidence of hemodynamic instability on physical exam. The patient did, however, experience urinary retention, constipation, and left hip pain with ambulation. Orthopedic follow-up and imaging revealed a posterior dislocation of the femoral component of her left hip prosthesis which subsequently required revision of both components of her left hip arthroplasty. The surgery proceeded under general anesthesia. There was no recurrence of bradycardia or transient asystole perioperatively, and no other complications were noted. She returned to the skilled nursing facility to complete her rehab. Orthopedic follow-up after hip arthroplasty revision surgery revealed the components to be in appropriate positions with minimal incisional pain. The patient reported progress with physical therapy and the ability to ambulate with her walker.

## 3. Discussion

The parasympathetic nervous system (PSNS) is comprised of cranial and sacral components. Cranial components originate from brainstem nuclei and leave the central nervous system (CNS) with cranial nerves, whereas sacral components originate from the spinal cord as the pelvic splanchnic nerves. The vagus nerve is particularly influential within the PSNS as it carries 75% of all parasympathetic fibers [6]. It originates at the medulla oblongata, projecting from the brain stem bilaterally as the right and left vagi bundled within each carotid sheath. The vagus consists of approximately 80% sensory afferent and 20% motor efferent fibers and demonstrates such widespread distribution throughout the body that its terminal targets are not completely known [7].

A well-described vagally-mediated reflex bradycardia occurs during laparoscopic surgery in response to increased intra-abdominal and intrapelvic pressures when a pneumoperitoneum is first created by the surgeon [8]. Parasympathetic sensory afferents from the abdominal viscera, peritoneum, and pelvic structures transmit impulses to the CNS via vagal and splanchnic nerve pathways where their synaptic connections distribute visceral information [9]. Vagal efferents from the Dorsal Motor Nucleus exit the medulla oblongata to synapse with the sinoatrial (SA) node where the most common side effect is bradycardia, however, other potentially fatal arrhythmias such as asystole, and even cardiac arrest may occur [9]. How or if parasympathetic sensory information transmitted via the pelvic splanchnic nerves contributes, either peripherally or centrally, toward vagally-mediated bradycardia remains unknown [7].

In 2002, Gallagher and Wilkinson published a small case series reporting severe intraoperative bradycardia/asystole during reaming (2 cases) and hammering (4 cases) of the femur in patients undergoing TKA [3]. During reaming, hydraulic pressure in the femoral medulla has been measured at 496 mmHg [10]. Gallagher and Wilkinson postulated this elevated intramedullary pressure indirectly causes a vagally-mediated reflex bradycardia when transmitted to intrapelvic and intra-abdominal viscera: the Gallagher–Wilkinson reflex [3]. Despite this dysrhythmia, all patients tolerated the surgical procedures without complications.

Joint arthroplasty is a relatively safe procedure with the incidence of cardiac-related complications such as cardiac arrest, myocardial infarction, or new onset arrhythmia reported at 0.2%–0.8% [11]. Advanced age and pre-existing cardiac disease were the highest risk factors for a major cardiac complication in patients undergoing THA procedures [11]. Intraoperative cardiac dysrhythmias have multiple etiologies which can include cardiopulmonary events such as myocardial ischemia/infarction or pulmonary/fat embolism. Our patient was at higher risk for perioperative cardiac events due to her age and pre-existing AF. A known complication of orthopedic surgery associated with intramedullary reaming includes fat embolism [12, 13]. However, both ischemic- and embolism-related causes of intraoperative dysrhythmias would be expected to induce more sustained myocardial depression than the brief and transient episodes our patient experienced [14]. Another rare cause of intraoperative asystole is due to bone cement implantation and resultant microemboli which can lead to both cardiac ischemia and pulmonary emboli [15–17], but the bradycardia and transient asystole observed in our patient occurred before bone cement was applied. The quick resolution of the patient's bradycardia and transient asystole immediately following the cessation of reaming, is most consistent with a vagal reflex response.

**Figure 2:**
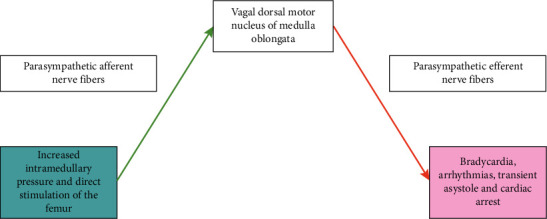
Schematic for a postulated direct mechanism of the Gallagher–Wilkinson reflex.

As previously discussed, the vagus nerve plays an important role in communication between higher neural circuitry and autonomic functions [7]. Various animal and clinical studies have suggested an association between the cholinergic system and bone [7, 18–23]. Parasympathetic nerves have been identified in both the axial and appendicular skeleton in mouse models [7, 19]. Additionally, immunohistochemical staining of trabecular bone in the distal femoral metaphysis demonstrated vesicular acetylcholine (ACh) transporter and skeletal innervation by the PSNS consisting of functional cholinergic nerve fibers, production of the neurotransmitter ACh, as well as bone cell expression of ACh receptors and its rate-limiting enzyme, acetylcholinesterase [19]. ACh produced in the bone also plays a role in the mobilization of hematopoietic stem cells from the bone marrow to the bloodstream [20, 21]. Studies suggest that cholinergic fibers innervate bone and promote bone formation via muscarinic ACh receptors [22]. In a nested case-control study, researchers showed that the use and treatment adherence to acetylcholinesterase inhibitors were associated with a decreased risk of osteoporotic fractures in elderly patients with Alzheimer's disease [23], which suggested that cholinergic fibers can impact bone remodeling in clinical data. The same group of researchers also showed an increase in bone density mass in patients treated with vagal nerve stimulation which demonstrates how direct electrical stimulation of the vagus nerve causes bone remodeling [18]. Based on preclinical and clinical research, we postulate that vagal afferent nerves are present in the bone axis, and stress from intramedullary reaming might directly activate the PSNS in the bone axis, causing vagally-mediated bradycardia and even transient asystole (Figure 2).

The Gallagher–Wilkinson reflex was also implicated in two other case reports of transient and reproducible bradycardia and asystole during reaming of the femoral canal [4, 5]. Like Gallagher and Wilkinson, the authors considered and discounted other potential physiologic causes such as microemboli or primary cardiac arrhythmia. The reproducible and transient nature of the bradycardic and asystolic events, associated with a specific trigger, more likely favored a vagally-mediated reflex. Current literature, however, does not completely delineate the extent of vagus nerve innervation. Studies indicate the presence of cholinergic fibers in the bone axis [19–22] and that stimulation of the vagus nerve can impact bone modulation in humans [18, 23]. The direct or indirect relationship of the Gallagher–Wilkinson reflex to vagus nerve stimulation requires additional research.

## 4. Conclusion

Previously offered explanations for the Gallagher–Wilkinson reflex described an indirect mechanism whereby high pressures applied to the femur are transmitted through the pelvis to intra-abdominal/intrapelvic organs thus triggering the vagally-mediated bradycardia. In light of studies which document parasympathetic innervation of bone, we offer the explanation of a direct mechanism whereby high pressures applied to the femur directly trigger the vagally-mediated reflex bradycardia. This explanation opens the possibility of intraoperative management using anticholinergics, such as glycopyrrolate or atropine, for patients without contraindications during orthopedic surgeries to prevent reflex bradycardia [24, 25]. An in-depth understanding of the underlying mechanisms of the Gallagher–Wilkinson reflex, particularly the role of parasympathetic innervation of bone in vagally-mediated bradycardia, could lead to more targeted intraoperative strategies and improved management.

## Data Availability

The data of this study are included within the article.

## References

[1] Shichman I., Roof M., Askew N. (2023). Projections and Epidemiology of Primary Hip and Knee Arthroplasty in Medicare Patients to 2040–2060. *JBJS Open Access*.

[2] Koessler M. J., Pitto R. P. (2001). Fat and Bone Marrow Embolism in Total Hip Arthroplasty. *Acta Orthopaedica Belgica*.

[3] Wilkinson D. J., Gallagher L. (2002). The Gallagher-Wilkinson Reflex. *Anaesthesia*.

[4] Martinez F. A., Dugdale E. M., Sims I. I. I., Charles R., Hofer R. E., Sems S. A. (2023). Intraoperative Dysrhythmias Cease after Venting during Intramedullary Nailing of an Impending Femur Fracture: A Case Report. *JBJS Case Connector*.

[5] Wooster B. M., Nickel B. T., Neumann J. A., Lindsay D. R., Wellman S. S. (2017). Reproducible Transient Asystolic Arrest during Intramedullary Reaming of the Femoral Canal: A Case Report. *Journal of Clinical Orthopaedics and Trauma*.

[6] Tindle J., Tadi P. (2022). *Neuroanatomy, Parasympathetic Nervous System*.

[7] Johnson R. L., Wilson C. G. (2018). A Review of Vagus Nerve Stimulation as a Therapeutic Intervention. *Journal of Inflammation Research*.

[8] Heyba M., Khalil A., Elkenany Y. (2020). Severe Intraoperative Bradycardia during Laparoscopic Cholecystectomy Due to Rapid Peritoneal Insufflation. *Case Reports in Anesthesiology*.

[9] Dabbous A. S., Baissari M. C., Nehme P. W., Esso J. J., Abu Leila M. (2014). Perioperative Reflex Bradycardia and Cardiac Arrest. *Middle East Journal of Anesthesiology*.

[10] Heim D., Schlegel U., Perren S. M. (1994). Intramedullary Pressure in Intramedullary Nailing of the Femur and Tibia. *Helvetica Chirurgica Acta*.

[11] Elsiwy Y., Jovanovic I., Doma K., Hazratwala K., Letson H. (2019). Risk Factors Associated with Cardiac Complication after Total Joint Arthroplasty of the Hip and Knee: A Systematic Review. *Journal of Orthopaedic Surgery and Research*.

[12] Rothberg D. L., Makarewich C. A. (2019). Fat Embolism and Fat Embolism Syndrome. *JAAOS-Journal of the American Academy of Orthopaedic Surgeons.*.

[13] Bulger E. M., Smith D. G., Maier R. V., Jurkovich G. J. (1997). Fat Embolism Syndrome: A 10-year Review. *Archives of Surgery*.

[14] Kalbas Y., Seaver T., Kumabe Y. (2022). Fat Embolism Syndrome in Patients with Bilateral Femur Fractures: A Systematic Review and Case Comparison. *OTA International*.

[15] Mudgalkar N., Ramesh K. V. (2011). Bone Cement Implantation Syndrome: A Rare Catastrophe. *Anesthesia: Essays and Researches*.

[16] Oancea A. F., Jigoranu R. A., Morariu P. C. (2023). Atrial Fibrillation and Chronic Coronary Ischemia: A Challenging Vicious Circle. *Life*.

[17] Mulpuru S. K., Rabinstein A. A., Asirvatham S. J. (2014). Atrial Fibrillation and Stroke: A Neurologic Perspective. *Cardiac Electrophysiology Clinics.*.

[18] Tamimi A., Tamimi F., Juweid M., Abdelkarim Al-Q. (2021). Could Vagus Nerve Stimulation Influence Bone Remodeling?. *Journal of Musculoskeletal and Neuronal Interactions*.

[19] Bajayo A., Bar A., Denes A. (2012). Skeletal Parasympathetic Innervation Communicates Central IL-1 Signals Regulating Bone Mass Accrual. *Proceedings of the National Academy of Sciences*.

[20] Fielding C., García-García A., Korn C., Stephen G. (2022). Cholinergic Signals Preserve Haematopoietic Stem Cell Quiescence during Regenerative Haematopoiesis. *Nature Communications*.

[21] García-García A., Korn C., García-Fernández M. (2019). Dual Cholinergic Signals Regulate Daily Migration of Hematopoietic Stem Cells and Leukocytes. *Blood*.

[22] Gadomski S., Fielding C., García-García A. (2022). A Cholinergic Neuroskeletal Interface Promotes Bone Formation during Postnatal Growth and Exercise. *Cell Stem Cell*.

[23] Tamimi I., Nicolau B., Eimar H. (2018). Acetylcholinesterase Inhibitors and the Risk of Osteoporotic Fractures: Nested Case-Control Study. *Osteoporosis International*.

[24] Gallanosa A., Stevens B. J., Hendrix J. M., Quick J. (2019). *Glycopyrrolate.* National Institutes of Health (NIH) Bookshelf. https://www.ncbi.nlm.nih.gov/books/NBK526035/.

[25] McLendon K., Preuss C. V. (2023). Atropine. National Library of Medicine. https://www.ncbi.nlm.nih.gov/books/NBK470551/.

